# Editorial: Non-coding RNA Regulation: Lessons from Model Organisms and Impact on Human Health

**DOI:** 10.3389/fgene.2016.00049

**Published:** 2016-03-31

**Authors:** Karim Mekhail

**Affiliations:** Department of Laboratory Medicine and Pathobiology, Faculty of Medicine, University of TorontoToronto, ON, Canada

**Keywords:** ncRNA, genetic models, humans, lncRNA, miRNA, neurodegeneration, inflammation, cancer

This research topic highlights the great interest and cautious optimism surrounding our constantly expanding understanding of the roles played by non-coding RNA (ncRNA) molecules in fundamental biology as well as in human health, aging, and disease.

In this topic, five review articles and one hypothesis/theory paper cover in breadth and depth the established, emerging, and controversial aspects of ncRNA biology. Claycomb discusses how we owe, at least in large part, today's broad interest in this area of biology to the discovery and characterization of RNA interference in the tiny soil worm *Caenorhabditis elegans*, a simple and elegant model organism that continues to be at the forefront of ncRNA research (Youngman and Claycomb). She also discusses how this worm provides a tractable genetic system in which researchers continue to study aging, epigenetic inheritance, and the role of ncRNA in such processes. Continuing with contributions from genetic model organisms to the field, Mekhail then discusses how yeast, fly, mouse, and human systems have helped uncover roles of ncRNA molecules in neural function and neurological disorders across the lifespan (Szafranski et al.). In particular, this article discusses the role of different classes of ncRNA including miRNA and lncRNA in healthy neural function and aging before highlighting how deleterious processes can also be triggered by ncRNA and/or its regulators in neurodegenerative diseases especially as we age. Suh then summarizes the rich connections between a great number of disease-linked miRNAs and the insulin growth factor 1 (IGF-1) signaling pathway, which regulates diverse processes from development to aging (Jung and Suh). Discussed connections between miRNAs and diseases including cancers, diabetes, and aging are astounding. This certainly highlights the therapeutic potential of miRNAs and miRNA-targeting nucleotides. Fish then discusses how networks of miRNAs and lncRNAs modulate NFkB signaling, a major driver of blood vessel inflammation (Cheng et al.). Such inflammation can start at a younger age but lead to heart attack and stroke later in life highlighting the therapeutic potential of ncRNA modulation to treat vascular inflammation and its associated complications across the lifespan. Mhlanga discusses how ncRNAs can be manipulated by pathogens such as the human immunodeficiency virus (HIV) (Barichievy et al.). This article reveals how ncRNA molecules are at the center of a power struggle between virus and host fighting for the regulatory potential of ncRNAs. Finally, Palazzo helps us take a step back and carefully assess the possibility that most ncRNA is junk transcriptional noise (Palazzo and Lee). Contrary to the first impression that this article is anti-ncRNA, this hypothesis and theory article should help the field better distinguish functional from junk ncRNA.

In conclusion, I thank the authors and my co-guest editor for their contributions to this research topic. I also extend my most sincere thanks to the tireless researchers in the ncRNA field for their dedication to decipher what continues to be one of the most fascinating areas of biology. In addition to revealing secrets of human health, disease, and aging, this field is helping us better understand the evolution of processes that are so vital to our existence as a human species. By uncovering these processes, we learn more about who we are in an effort to extend our health span.

## Author contributions

The author confirms being the sole contributor of this work and approved it for publication.

## Funding

KM is supported by the Canadian Institutes of Health Research Open Operating Grant Program and the Ontario Early Researcher Award. KM holds the Canada Research Chair in Spatial Genome Organization.

### Conflict of interest statement

The author declares that the research was conducted in the absence of any commercial or financial relationships that could be construed as a potential conflict of interest.

